# 503. Antibiotic Mobile Application ABxSG - An Innovative and Effective Antibiotic Stewardship Tool that Improves Antibiotic Use and Reduces Healthcare Costs

**DOI:** 10.1093/ofid/ofae631.155

**Published:** 2025-01-29

**Authors:** Shena Yun Chun Lim, Lai Wei Lee, Peijun Yvonne Zhou, Boon San Teoh, Daphne Yah Chieh Yii, Jia Le Lim, Jun Jie Tan, Kai Chee Hung, Li Wen Loo, Nathalie Chua, Narendran Koomanan, Yi Xin Liew, Li Xuan Trevina Lee, YiBo Wang, Winnie Lee, Cherie Si Le Gan, Siew Yee Thien, Benjamin Pei Zhi Cherng, Maciej Piotr Chlebicki, Lay Hoon Andrea Kwa, Shimin Jasmine Chung

**Affiliations:** Singapore General Hospital, Singapore, Singapore; Singapore General Hospital, Singapore, Singapore; Singapore General Hospital, Singapore, Singapore; Singapore General Hospital, Singapore, Singapore; Singapore General Hospital, Singapore, Singapore; Singapore General Hospital, Singapore, Singapore; Singapore General Hospital, Singapore, Singapore; Singapore General Hospital, Singapore, Singapore; Singapore General Hospital, Singapore, Singapore; Singapore General Hospital, Singapore, Singapore; Singapore General Hospital, Singapore, Singapore; Singapore General Hospital, Singapore, Singapore; Singapore General Hospital, Singapore, Singapore; Singapore General Hospital, Singapore, Singapore; Singapore General Hospital, Singapore, Singapore; Singapore General Hospital, Singapore, Singapore; Singapore General Hospital, Singapore, Singapore; Singapore General Hospital, Singapore, Singapore; Singapore General Hospital, Singapore, Singapore; Singapore General Hospital, Singapore, Singapore; Singapore General Hospital, Singapore, Singapore

## Abstract

**Background:**

At Singapore General Hospital (SGH), 50% of inpatients receive ≥1 antibiotic & 30% are inappropriate. In an in-house survey in 2020, doctors opined that access to hospital antibiotic guidelines on their mobile devices is time-saving and improves antibiotic use. A mobile application, ABxSG, was developed and launched in Mar 2023, providing a one stop platform for hospital guidelines, educational materials on disease management and curated medical calculators [Figure 1]. At 9 months post launch, there were 2,400 ABxSG users.

**Figure 1.**
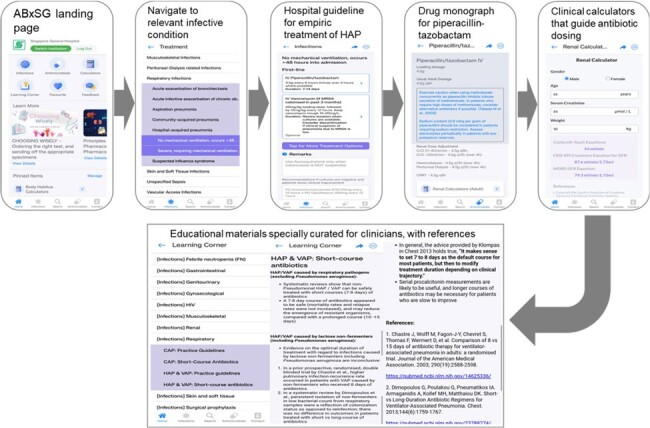
Illustration of the features of ABxSG mobile application that aids clinicians in managing a hospital acquired pneumonia

**Methods:**

An interrupted time series analysis using autoregressive integrated moving average model was conducted to evaluate the impact of ABxSG on the proportion of inpatients on antibiotics and the number of pharmacist interventions made pertaining to antibiotic related monitoring parameters to ensure efficacy/safety. The time period analyzed included 1 year pre- (Apr 22-Mar 23) & 9 month post-(Apr 23-Dec 23) ABxSG.

**Figure 2.**
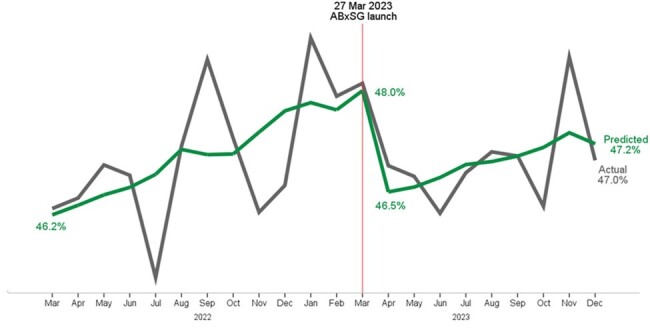
The actual & predicted proportion of inpatients on antibiotics decreased after the launch of ABxSG

**Results:**

The proportion of inpatients on antibiotic was rising 0.15%/month pre-ABxSG & slowed down post-ABxSG to 0.11%/month (p=0.67) [Fig. 2]. Notably, there was an immediate reduction of 1.5% post ABxSG (p< 0.05). At 9 months, there was a predicted 2.83% reduction (*p*= 0.09), translating to 600 antibiotic free days a month (with an estimated savings of ∼USD$37,000 in drug cost and 540 nursing hours a month).

The rising trend in number of interventions for antibiotic related monitoring parameters pre-ABxSG was reversed post-ABxSG (*p*< 0.05) [Fig. 3]. At 9 months, there was a predicted ∼37 less interventions a month (p< 0.05), a nearly 50% reduction from pre-ABxSG. This reduction translates to a saving of 23 pharmacist hours a month.

**Figure 3.**
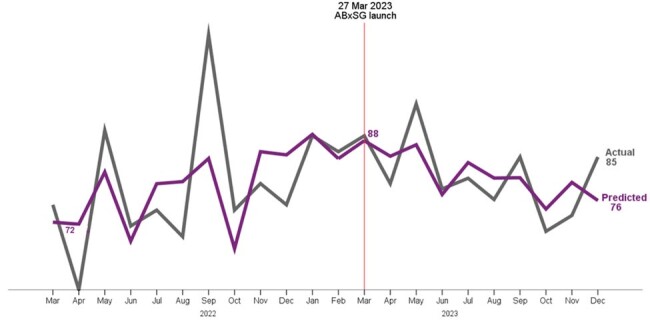
The actual & predicted number of interventions for antibiotic related monitoring parameters by inpatient pharmacists decreased after launch of ABxSG

**Conclusion:**

ABxSG reduced the proportion of patients on antibiotics and the number of pharmacist interventions for antibiotic related monitoring parameters. The antibiotic mobile application is an innovative digital solution to engage and empower prescribers to use antibiotics appropriately. Additionally, it is an aid for healthcare institutions where there is a shortage of pharmacists to provide close oversight on antibiotic use. With improved antibiotic prescribing, ABxSG can potentially reduce healthcare costs in the long run.

**Disclosures:**

**All Authors**: No reported disclosures

